# Elevated CO_2_ as a driver of global dryland greening

**DOI:** 10.1038/srep20716

**Published:** 2016-02-12

**Authors:** Xuefei Lu, Lixin Wang, Matthew F. McCabe

**Affiliations:** 1 Department of Earth Sciences, Indiana University-Purdue University Indianapolis (IUPUI), Indianapolis, USA; 2 Division of Biological and Environmental Sciences and Engineering, King Abdullah University of Science and Technology, Thuwal, Saudi Arabia

## Abstract

While recent findings based on satellite records indicate a positive trend in vegetation greenness over global drylands, the reasons remain elusive. We hypothesize that enhanced levels of atmospheric CO_2_ play an important role in the observed greening through the CO_2_ effect on plant water savings and consequent available soil water increases. Meta-analytic techniques were used to compare soil water content under ambient and elevated CO_2_ treatments across a range of climate regimes, vegetation types, soil textures and land management practices. Based on 1705 field measurements from 21 distinct sites, a consistent and statistically significant increase in the availability of soil water (11%) was observed under elevated CO_2_ treatments in both drylands and non-drylands, with a statistically stronger response over drylands (17% vs. 9%). Given the inherent water limitation in drylands, it is suggested that the additional soil water availability is a likely driver of observed increases in vegetation greenness.

Defined broadly as zones where mean annual precipitation is less than two-thirds of potential evaporation, drylands are critically important systems^1^,^2^,^3^ and represent the largest terrestrial biome on the planet^4^. Climate change, increasing populations and resulting anthropogenic effects are all expected to impact dryland regions over the coming decades^5^. Considering that approximately 90% of the more than 2 billion people living in drylands^6^ are geographically located within developing countries^2^, improved understanding of these systems is an international imperative. Recent regional scale analyses using satellite based vegetation indices such as the Normalized Difference Vegetation Index (NDVI), have found extensive areas of “greening” in dryland areas of the Mediterranean^7^, the Sahel^8^, the Middle East^9^ and Northern China^10^, as well as greening trends in Mongolia and South America^11^. More recently, a global synthesis over the period from 1982–2007 that used an integrated NDVI and annual rainfall, showed an overall “greening-up” trend over the Sahel belt, Mediterranean basin, China-Mongolia region and the drylands of South America^12^.

To better predict system responses to possible climate changes, it is necessary to understand the drivers behind the observed greening response. Several mechanisms may contribute to the apparent trends in vegetation greenness. For example, increasing rainfall is one obvious driver of change, with a number of studies establishing a positive relationship between NDVI and precipitation^8^,^12^. However, rainfall does not explain the observed trends at a global scale. Indeed, there are regions where greening occurs in the absence of any observed rainfall increases^12^. Likewise, there are areas where a significant rainfall increase occurs without a corresponding change in greening^12^. In addition, even in those regions experiencing concurrent greening and rainfall increase (such as in the African Sahel), removing the effects of rainfall from the NDVI time series does not completely remove the NDVI residual, indicating that the vegetation greening in the Sahel may be attributable to other factors^8^. Changes in land use or the implementation of improved management practices may also impact upon vegetation in certain areas, such as the observed agricultural expansions in Australia’s Murray-Darling basin, the Middle East and southwest United States, tree plantations in west China^13^, as well as grazing practices triggering changes in plant community composition in South Africa. Greening can also result from variations in species composition (e.g., exotic species invasion in many drylands^14^). However, similar to rainfall changes, human-induced factors and species composition changes are more likely to be an important local driver impacting vegetation response. As vegetation greening has been observed across all drylands, discriminating the influence of a potential global driver that is enhanced or suppressed by local scale factors, is one of the goals of this work.

To this end, we hypothesize that higher levels of atmospheric CO_2_ concentration are a key driver of the observed dryland greening, through an impact on plant water savings and consequent available soil water increase. A novel modeling framework introduced by Donohue *et al*.^15^, described higher vegetation water use efficiency (*WUE*) under CO_2_ enrichment, with the authors using this mechanism to explain increases in maximum vegetation cover in warm and dry environments. The hypothesis developed in this study implies that the greening in global drylands is a response to higher CO_2_ levels increasing the available soil water. The hypothesis is based on increasing atmospheric CO_2_ inducing decreases in plant stomatal conductance and enhancing vegetation *WUE*^15^,^16^. Higher *WUE* encourages increased soil water under the same productivity levels. Since soil water is a limiting factor in dryland vegetation growth and function^17^, any increase in available soil water is expected to enhance plant growth and greening.

Here we attempt to examine this hypothesis using a data driven meta-analytic approach. One of the key aims of this work is not just to identify the potential contribution of CO_2_ to observed changes in global greening, but also to identify different soil water responses that might be occurring within dryland and non-dryland systems. Understanding the varying interactions between soil water and vegetation under CO_2_ enrichment between dryland and non-dryland systems would significantly increase our capacity to predict vegetation response to future climatic changes, as dynamic vegetation responses often pose large uncertainty in global models.

## Results and Discussion

**Figure 1:**
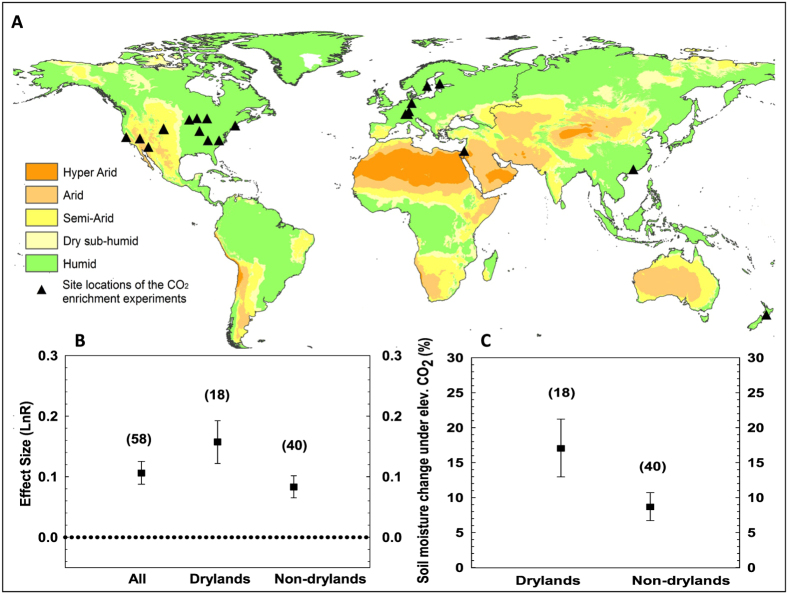
Global climate map and a comparison of mean effect size and soil water response under elevated CO_2_. **(A**) Site locations of the CO_2_ enrichment experiments together with globally distributed climate zones based on a standard aridity index formulation (precipitation/potential evapotranspiration); (**B**) Mean effect size of soil water content under elevated CO_2_ for the entire data set, under dryland and non-dryland regimes. The effect size was calculated as the natural log of the magnitude of an experimental treatment mean (the soil water under elevated CO_2_) relative to the control treatment mean (the soil water under ambient CO_2_); The dashed line indicates the threshold of statistically significant CO_2_ effect on soil moisture. The effect is positive when above the line and vice versa. (**C**) Enhancement of soil water content under elevated CO_2_ for dryland versus non-dryland regimes. The number of cases is shown in brackets. Error bars are bootstrapped confidence intervals (*CI*). All the statistics are significant at *P* < 0.05. The map was generated using ArcGIS for Desktop 10.3.1 (http://www.arcgis.com).

In order to test our hypothesis and to evaluate the soil water response differences occurring within dryland and non-dryland systems, a total of 45 studies from 8 countries (yielding 1705 measurements from 21 distinct sites), were included in the meta-analysis (Fig. 1A, Supplementary Table S1, Supplementary Appendix S1). The meta-analysis revealed that increasing atmospheric CO_2_ to between 1.2 to 2.0 times the ambient CO_2_ level has a positive effect on soil water content, indicated by the fact that the effect size was greater than zero in both drylands and non-drylands (Fig. 1B). When considering the entire data set, higher CO_2_ levels resulted in an 11% increase in soil water content across all systems (Fig. 1B). Importantly, the analysis revealed that elevated CO_2_ significantly enhanced soil water levels in drylands more so than it did in non-drylands (*P* < 0.05, Fig. 1C), with soil water content increasing by 9% in non-drylands compared to 17% in drylands (*P* < 0.05, Fig. 1C). According to our meta-analysis data set, the mean soil water content was 11.6% under the ambient CO_2_ level in drylands, while it was 24.1% in non-drylands. Based on the meta-analysis results, the enhanced CO_2_ level would result in a 1.9% absolute soil moisture change in drylands and 2.2% change in non-drylands. Although the absolute change of soil moisture in drylands is comparable to that in non-drylands, studies have shown that even small change of soil moisture in drylands could be significant enough to cause large changes in vegetation productivity^18^. The CO_2_ induced soil water increase seems contrary to the conventional understanding that any additional soil water should be transpired or evaporated in drylands, as water is a limiting resource. However, similar responses have been observed across many individual studies (Supplementary Table S1) and are apparent in our global synthesis at both dryland and non-dryland sites, highlighting the strong role vegetation plays in the soil water balance^17^. Importantly, the observed response lends weight to the hypothesis that any additional soil water in the root zone is then available to facilitate vegetation growth and greening under enhanced atmospheric CO_2_. Determining the mechanisms of stronger soil water responses in drylands requires further investigation, since it is generally thought that elevated CO_2_ has a smaller effect on stomatal response during dry periods or under extreme drought^19^.

The direct effects of elevated CO_2_ on photosynthesis can act to increase plant productivity through the alleviation of any carbon limitation^20^. However, CO_2_ is not a limiting factor in most drylands, where productivity is governed mainly by water and nutrient constraints^21^. Assuming that a direct CO_2_ effect occurs through the alleviation of carbon limitation in both dryland and non-dryland ecosystems, as shown earlier, our analysis has demonstrated that the indirect soil water response to elevated CO_2_ levels is 89% higher in drylands (*P* < 0.05, Fig. 1C), indicating that factors other than a direct CO_2_ effect play a role in increasing plant productivity in dryland systems.

**Figure 2:**
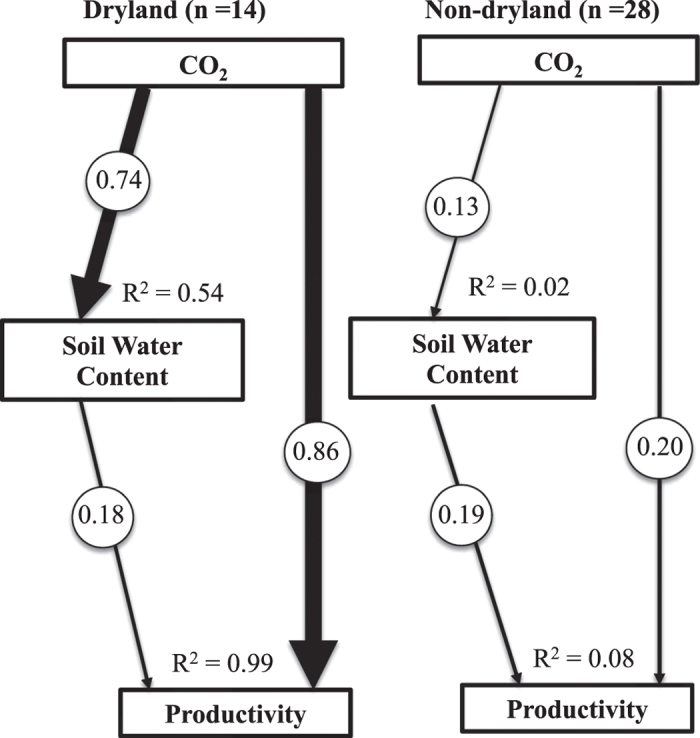
Structural equation modeling of direct and indirect effects of CO_2_ enrichment on vegetation productivity for both drylands and non-drylands. The number of cases is shown in brackets. Arrow thickness is proportional to path coefficient.

To explore this idea further, a SEM approach^22^ was used to test the relative importance of direct (increased CO_2_ removing any carbon limitation) versus indirect (i.e., increased CO_2_ increasing soil water content) links between CO_2_ enrichment and vegetation productivity for both drylands and non-drylands. SEM results show that the CO_2_ effect on productivity was stronger for both direct effects on growth (path coefficients = 0.86 for drylands and 0.2 for non-drylands) and indirect effects on soil water content (path coefficients = 0.74 for drylands and 0.13 for non-drylands) (Fig. 2), providing additional support that CO_2_ induced soil moisture increases are important in drylands.

**Figure 3:**
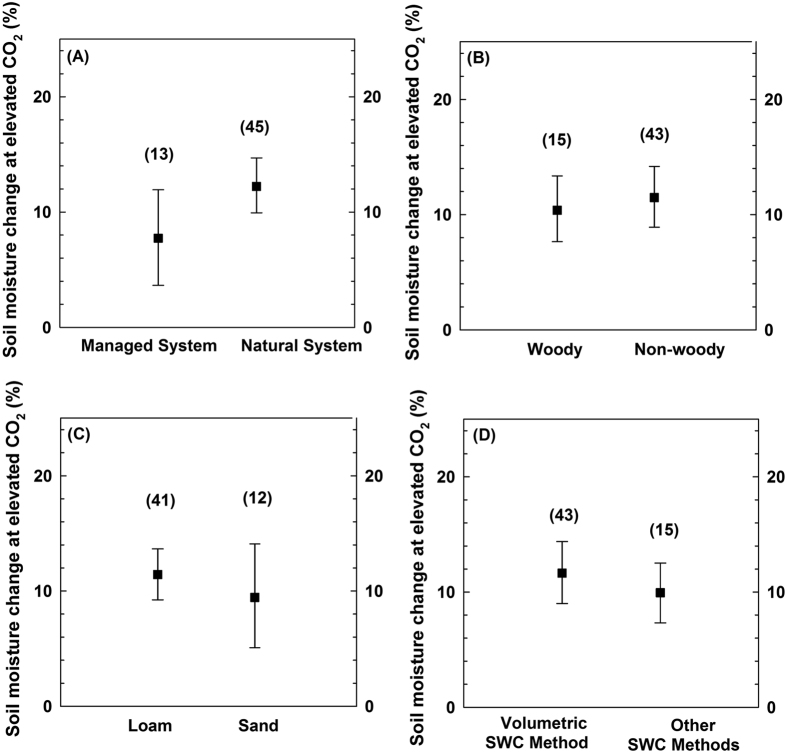
Enhancement of soil water content for elevated CO_2_ levels (A) under different management systems; (B) under different vegetation types; and (C) under different soil texture; and (D) using results from different soil water content (SWC) measurement methods (volumetric method, gravimetric method, etc., Extended Table 1). The number of cases is shown in brackets. Error bars are bootstrapped confidence intervals (*CI*). All the statistics are not significant at *P* > 0.05.

There are other variables that could affect the interaction between soil water content and elevated CO_2_ level, including soil texture, vegetation type and system type. However, with the protocols developed in this exercise, the meta-analysis shows no evidence for any significant effects of these on soil water under higher CO_2_ levels (Fig. 3A–C). In addition to accounting for the potential influence of other factors on vegetation response, the use of different methodologies to quantify soil water content has the capacity to influence the interpretation of results. To test any introduced methodological bias, we compared the results of studies reporting volumetric water content (the predominant unit used in the studies included in our analysis) and results using techniques such as gravimetric water content. The meta-analysis results were consistent between the different approaches (Fig. 3D).

**Figure 4:**
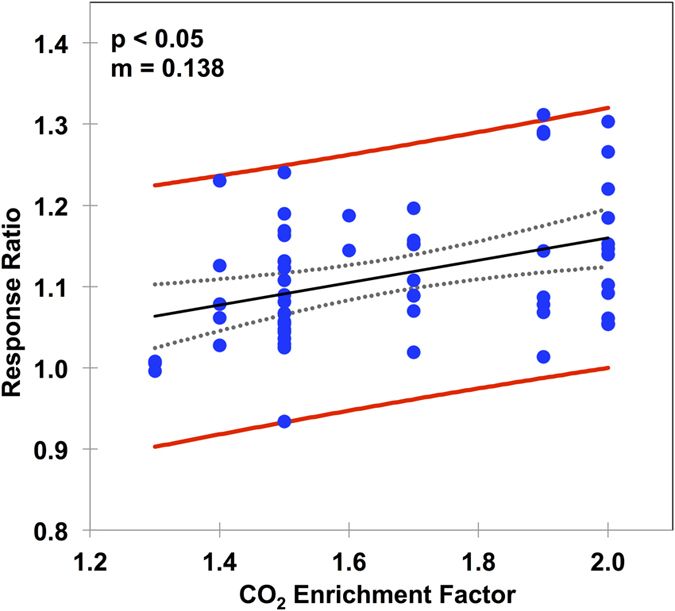
Sensitivity of the soil water response to CO_2_ enrichment for the entire data set. The response index was calculated as the soil water content under elevated CO_2_ divided by the soil water content under ambient CO_2_. The closed circles are the observations, with the solid black line providing a linear regression. The red lines represent the 95% confidence intervals of the observations and the dashed grey lines represent the 95% confidence interval of the model. m is the slope of the regression line.

To date, the global average concentration of CO_2_ in the atmosphere has increased by nearly 27% (from 315 ppm to approximately 400 ppm) over the period 1960–2015^23^, with the expectation of a continued rise into the 21st century. To establish the validity of using results from higher CO_2_ enrichment experiments (1.2 to 2.0 times ambient atmospheric CO_2_) to explain the soil water-vegetation responses observed under current CO_2_ levels, we examined the sensitivity of soil water change to varying levels of CO_2_ using a regression analyses. Using the global meta-analysis data, a significant positive change in soil water along the CO_2_ enrichment gradient was determined (*P* < 0.05, Fig. 4), supporting the CO_2_ enrichment effect on soil water. At the same time, the rate of change was low (slope = 0.138, Fig. 4), indicating that soil water changes in response to CO_2_ are comparable between higher CO_2_ enrichment levels (1.2–2.0) and currently observed CO_2_ enrichment (~1.27). The stability of the rate of change justifies using higher CO_2_ enrichment levels to interpret soil water responses to currently observed CO_2_ enrichment.

As noted earlier, increasing CO_2_ is not the only potential driver of changes in vegetation response. Temperature increases could also affect dryland plant productivity and greenness. Studies on the impact of concurrent CO_2_ and temperature increase upon *WUE* have found that *WUE* is substantially increased by elevated CO_2_, despite a significant increase in air temperature, because the increase in leaf temperature is not significantly different between CO_2_ treatments due to evaporative cooling of the leaf 24. In addition, none of the CO_2_ enrichment studies used in this data synthesis have a concurrent temperature treatment operating, indicating that temperature is not a confounding factor for our main conclusion. At the same time, we argue temperature is an important factor to constrain the degree of CO_2_ induced greening due to its direct and negative impact on *WUE* and vegetation phenology. For example, an experiment over an agricultural field in a semi-arid region of China showed that *WUE* decrease by 7.3% with a mean daily temperature increase of 1.2 °C^25^. In some Mediterranean-type ecosystems such as annual-dominated California grasslands, warming has accelerated the decline of canopy greenness because the effects of reduced transpiration losses push the canopy to an earlier senescence^26^. These facts indicate that the positive effect of CO_2_ induced water savings may eventually be offset by the negative effect of CO_2_ induced temperature increases when the temperature increase crosses a certain threshold. Further understanding of this complex feedback process is required.

## Conclusions

Dryland greening presents something of a paradox in our intuitive understanding of plant-water-CO_2_ interactions. Combining our meta-analysis results and early work, it illustrates that higher concentrations of atmospheric CO_2_ induce plant water saving and that consequent available soil water increases are a likely driver of the observed greening phenomena. Our results support recent modeling work showing higher vegetation *WUE* and higher maximum vegetation cover under CO_2_ enrichment in warm and dry environments^15^. The time scale of the CO_2_ enrichment effect on greening may have potential implications on global carbon budgets, as drylands have been found to be significant players in modulating the inter-annual variability of carbon cycling^27^. By identifying the contributing mechanisms that result in vegetation greenness, our findings provide important insights into plant-water interactions. Predicting system level response to future climatic and/or anthropogenic perturbations in dryland systems remains a critically important but under-investigated area of inquiry.

## Methods

Our study is based on an analysis of data obtained from field experiments in which changes in soil water were measured under elevated atmospheric CO_2_ concentrations using a Free-Air CO_2_ Enrichment (FACE) facility or open top chamber. To collect the data required in the meta-analysis, a comprehensive literature search using the terms ‘CO_2_ enrichment’, ‘soil moisture’, ‘FACE’, ‘open top chamber’ and ‘growth chamber’ was conducted across Thomson Reuters Web of Science and Google Scholar databases. All of the field data used in this study was derived from *in-situ* field experiments that examined soil water responses to both ambient and elevated atmospheric CO_2_ levels.

A rigorous procedure was employed to ensure the independence of each data entry, avoiding over-representation of any particular study and reducing publication bias. For instance, in cases where data were collected over consecutive years, but using identical treatments with the same soil texture and vegetation cover, data were averaged and only a single entry from that study was used in the meta-analysis. In cases where different types of vegetation cover or soil texture were used, or where the same experiment was carried out under different treatments (e.g., nitrogen addition vs. control), data were treated as separate contributions (Supplementary Table S1). When soil water content was measured at multiple depths, only the top 0–25 cm measurements were used in the meta-analysis. We focus on soil water content from the growing season only, since this is the period with the closest interaction between vegetation and soil water. Changing the data length to include the entire monitoring period of each individual study yielded similar results, as shown in Supplementary Figure S1.

The Meta-Win 2.0 software^28^ was used to perform statistical analysis on results. In order to include those studies that did not adequately report sample sizes or standard deviations, we conducted an unweighted analysis using the log response ratio (ln*R*) to calculate bootstrapped confidence limits^28^. Elevated CO_2_ was considered to have a significant effect on soil water content if the bootstrap confidence interval did not overlap with zero^28^. The CO_2_ response of two groups was considered significantly different if their bootstrap confident intervals did not overlap. A statistical significance level of *P* < 0.05 was used.

A structural equation model (SEM)^22^ was also employed to test the relative importance of direct versus indirect linkages between CO_2_ enrichment and vegetation productivity for both drylands and non-drylands using all the available data. SEM statistics were calculated using International Business Machines (IBM) SPSS AMOS version 22 (AMOS Development Corp. Meadville, PA). We used a maximum likelihood based goodness-of-fit test to assess the degree of accord between observed and predicted covariance structures. Because our models were saturated, i.e., all possible pathways between all variables were accounted for, we could not test the significance of our models^22^. The calculated path coefficients are based on the amount of variance explained in the response variables and they represent relative strengths of the specific pathways. R^2^ values represent the total variances explained by all of the contributing variables.

To test the soil water response under different climate regimes, we classified the study locations as “dryland vs. non-dryland” based on an aridity index database (Fig. 1). Following the United Nations Environment Program (UNEP) terminology, drylands are defined as regions where the Aridity Index (AI) is smaller than 0.65, with AI expressed as the ratio of mean annual precipitation to mean annual potential evapotranspiration. In addition to climatic regimes, a number of other factors might affect the response of the available soil water under CO_2_ enrichment. These include the system type, vegetation type and soil texture. We classified the system types as “natural vs. managed” by defining agriculture as a managed ecosystem and the remainder (i.e., forest and grassland) as natural systems (Fig. 3A). Similarly, vegetation was discriminated into “woody vs. non-woody”, with the latter comprising grassland and cropland (Fig. 3B). Soil texture was grouped into two classes based upon the United States Department of Agriculture (USDA) soil texture triangle: (1) Sand, which includes sand and loamy sand; and (2) Loam, which includes loam, clay loam, silt loam, sandy loam, and silty clay loam (Fig. 3C). To test any potential introduced methodological bias, we compared the results of studies reporting volumetric water content (the predominant unit used in the studies comprising our synthesis) and results using other techniques such as gravimetric water content (Fig. 3D).

## Additional Information

**How to cite this article**: Lu, X. *et al*. Elevated CO_2_ as a driver of global dryland greening. *Sci. Rep*. **6**, 20716; doi: 10.1038/srep20716 (2016).

## Supplementary Material

Supplementary Information
