# Supplementary material for: Elevated CO2 as a driver of global dryland greening
Source: Sci Rep. 2016 Feb 12;6:20716. doi: 10.1038/srep20716 (PMC4751615; doi:10.1038/srep20716)
Supplement: Supplementary Information [file srep20716-s1.pdf]

Is elevated CO_2 a driver of global dryland greening?

Xuefei Lu[1], Lixin Wang[1]*, Matthew F. McCabe[2]

[1]Department of Earth Sciences, Indiana University-Purdue University Indianapolis (IUPUI), Indianapolis, USA,
[2]Division of Biological and Environmental Sciences and Engineering, King Abdullah University of Science and Technology, Thuwal, Saudi Arabia

Supplementary Appendix S1. List of references used to collect database for this study

1. Adair, E.C., Reich, P.B., Trost, J.J. & Hobbie, S.E. Elevated CO_2 stimulates grassland soil respiration by increasing carbon inputs rather than by enhancing soil moisture. Global Change Biology 17, 3546-3563 (2011).

2. Allard, V. et al. Increased quantity and quality of coarse soil organic matter fraction at elevated CO_2 in a grazed grassland are a consequence of enhanced root growth rate and turnover. Plant and Soil 276, 49-60 (2005).

3. Ambus, P. & Robertson, G.P. Fluxes of CH_4 and N_2O in aspen stands grown under ambient and twice-ambient CO_2. Plant and Soil 209, 1-8 (1999).

4. Arnone, J.A. & Bohlen, P.J. Stimulated N_2O flux from intact grassland monoliths after two growing seasons under elevated atmospheric CO_2. Oecologia 116, 331-335 (1998).

5. Bader, M., Hiltbrunner, E. & Korner, C. Fine root responses of mature deciduous forest trees to free air carbon dioxide enrichment (FACE). Functional Ecology 23, 913-921 (2009).

6. Baggs, E.M., Richter, M., Hartwig, U.A. & Cadisch, G. Nitrous oxide emissions from grass swards during the eighth year of elevated atmospheric pCO(2) (Swiss FACE). Global Change Biology 9, 1214-1222 (2003).

7. Baggs, E.M. & Blum, H. CH_4 oxidation and emissions of CH_4 and N_2O from Lolium perenne swards under elevated atmospheric CO_2. Soil Biology & Biochemistry 36, 713-723 (2004).

8. Burkart, S., Manderscheid, R. & Weigel, H.J. Interactive effects of elevated atmospheric CO_2 concentrations and plant available soil water content on canopy evapotranspiration and conductance of spring wheat. European Journal of Agronomy 21, 401-417 (2004).

9. Catovsky, S. & Bazzaz, F.A. Elevated CO_2 influences the responses of two birch species to soil moisture: implications for forest community structure. Global Change Biology 5, 507-518 (1999).

10. Carrillo, Y., Pendall, E., Dijkstra, F.A., Morgan, J.A. & Newcomb, J.M. Response of soil organic matter pools to elevated CO_2 and warming in a semi-arid grassland. Plant and Soil 347, 339-350 (2011).

11. Conley, M.M. et al. CO_2 enrichment increases water-use efficiency in sorghum. New Phytologist 151, 407-412 (2001).

12. Craine, J.M., Wedin, D.A. & Reich, P.B. The response of soil CO_2 flux to changes in atmospheric CO_2, nitrogen supply and plant diversity. Global Change Biology 7, 947-953 (2001).

13. Decock, C. et al. Elevated CO_2 and O_3 modify N turnover rates, but not N_2O emissions in a soybean agroecosystem. Soil Biology & Biochemistry 51, 104-114 (2012).

14. Deng, Q. et al. Responses of soil respiration to elevated carbon dioxide and nitrogen addition in young subtropical forest ecosystems in China. Biogeosciences 7, 315-328 (2010).

15. Dijkstra, F.A. et al. Contrasting effects of elevated CO_2 and warming on nitrogen cycling in a semiarid grassland. New Phytologist 187, 426-437 (2010).

16. Dijkstra, F.A., Morgan, J.A., Follett, R.F. & Lecain, D.R. Climate change reduces the net sink of CH_4 and N_2O in a semiarid grassland. Global Change Biology 19, 1816-1826 (2013).

17. Dubbs, L.L. & Whalen, S.C. Reduced net atmospheric CH_4 consumption is a sustained response to elevated CO_2 in a temperate forest. Biology and Fertility of Soils 46, 597-606 (2010).

18. Edwards, G.R., Clark, H. & Newton, P.C.D. The effects of elevated CO_2 on seed production and seedling recruitment in a sheep-grazed pasture. Oecologia 127, 383-394 (2001).

19. Ellsworth, D.S. CO_2 enrichment in a maturing pine forest: are CO_2 exchange and water status in the canopy affected? Plant Cell and Environment 22, 461-472 (1999).

20. Erbs, M., Franzaring, J., Hogy, P. & Fangmeier, A. Free-air CO_2 enrichment in a wheat-weed assembly - effects on water relations. Basic and Applied Ecology 10, 358-367 (2009).

21. Fatichi, S. & Leuzinger, S. Reconciling observations with modeling: The fate of water and carbon allocation in a mature deciduous forest exposed to elevated CO_2. Agricultural and Forest Meteorology 174, 144-157 (2013).

22. Field, C.B., Lund, C.P., Chiariello, N.R. & Mortimer, B.E. CO_2 effects on the water budget of grassland microcosm communities. Global Change Biology 3, 197-206 (1997).

23. Grunzweig, J.M. & Korner, C. Growth, water and nitrogen relations in grassland model ecosystems of the semi-arid Negev of Israel exposed to elevated CO_2. Oecologia 128, 251-262 (2001).

24. Kettunen, R., Saarnio, S., Martikainen, P. & Silvola, J. Elevated CO_2 concentration and nitrogen fertilisation effects on N_2O and CH_4 fluxes and biomass production of Phleum pratense on farmed peat soil. Soil Biology & Biochemistry 37, 739-750 (2005).

25. Leakey, A.D.B. et al. Photosynthesis, productivity, and yield of maize are not affected by open-air elevation of CO_2 concentration in the absence of drought. Plant Physiology 140, 779-790 (2006).

26. Lecain, D.R., Morgan, J.A., Mosier, A.R. & Nelson, J.A. Soil and plant water relations determine photosynthetic responses of C_3 and C_4 grasses in a semi-arid ecosystem under elevated CO_2. Annals of Botany 92, 41-52 (2003).

27. LeCain, D.R., Morgan, J.A., Hutchinson, G.L., Reeder, J.D. & Dijkstra, F.A. Interactions between elevated atmospheric CO_2 and defoliation on North American rangeland plant species at low and high N availability. Grass and Forage Science 67, 350-360 (2012).

28. Leuzinger, S. & Korner, C. Water savings in mature deciduous forest trees under elevated CO_2. Global Change Biology 13, 2498-2508 (2007).

29. Liu, J.X. et al. CO_2 enrichment increases nutrient leaching from model forest ecosystems in subtropical China. Biogeosciences 5, 1783-1795 (2008).

30. Marhan, S., Kandeler, E., Rein, S., Fangmeier, A. & Niklaus, P.A. Indirect effects of soil moisture reverse soil C sequestration responses of a spring wheat agroecosystem to elevated CO_2. Global Change Biology 16, 469-483 (2010).

31. Matamala, R. & Schlesinger, W.H. Effects of elevated atmospheric CO_2 on fine root production and activity in an intact temperate forest ecosystem. Global Change Biology 6, 967-979 (2000).

32. McCarthy, H.R. et al. Re-assessment of plant carbon dynamics at the Duke free-air CO_2 enrichment site: interactions of atmospheric CO_2 with nitrogen and water availability over stand development. New Phytologist 185, 514-528 (2010).

33. McLain, J.E.T. & Ahmann, D.M. Increased moisture and methanogenesis contribute to reduced methane oxidation in elevated CO_2 soils. Biology and Fertility of Soils 44, 623-631 (2008).

34. Morgan, J.A., LeCain, D.R., Mosier, A.R. & Milchunas, D.G. Elevated CO_2 enhances water relations and productivity and affects gas exchange in C_3 and C_4 grasses of the Colorado shortgrass steppe. Global Change Biology 7, 451-466 (2001).

35. Mosier, A.R., Morgan, J.A., King, J.Y., LeCain, D. & Milchunas, D.G. Soil-atmosphere exchange of CH_4, CO_2, NO_x, and N_2O in the Colorado shortgrass steppe under elevated CO_2. Plant and Soil 240, 201-211 (2002).

36. Nelson, J.A. et al. Elevated CO_2 increases soil moisture and enhances plant water relations in a long-term field study in semi-arid shortgrass steppe of Colorado. Plant and Soil 259, 169-179 (2004).

37. Nendel, C. et al. Testing different CO_2 response algorithms against a FACE crop rotation experiment. Njas-Wageningen Journal of Life Sciences 57, 17-25 (2009).

38. Newton, P.C.D., Carran, R.A. & Lawrence, E.J. Reduced water repellency of a grassland soil under elevated atmospheric CO_2. Global Change Biology 10, 1-4 (2004).

39. Niklaus, P.A., Spinnler, D. & Korner, C. Soil moisture dynamics of calcareous grassland under elevated CO_2. Oecologia 117, 201-208 (1998).

40. Pataki, D.E. et al. Water use of two Mojave Desert shrubs under elevated CO_2. Global Change Biology 6, 889-897 (2000).

41. Pendall, E. et al. Elevated atmospheric CO_2 effects and soil water feedbacks on soil respiration components in a Colorado grassland. Global Biogeochemical Cycles 17 (2003).

42. Pregitzer, K., Loya, W., Kubiske, M. & Zak, D. Soil respiration in northern forests exposed to elevated atmospheric carbon dioxide and ozone. Oecologia 148, 503-516 (2006).

43. Sindhoj, E. et al. Root dynamics in a semi-natural grassland in relation to atmospheric carbon dioxide enrichment, soil water and shoot biomass. Plant and Soil 223, 253-263 (2000).

44. Volk, M., Niklaus, P.A. & Korner, C. Soil moisture effects determine CO_2 responses of grassland species. Oecologia 125, 380-388 (2000).

45. Wall, G.W. et al. Elevated atmospheric CO_2 improved Sorghum plant water status by ameliorating the adverse effects of drought. New Phytologist 152, 231-248 (2001).

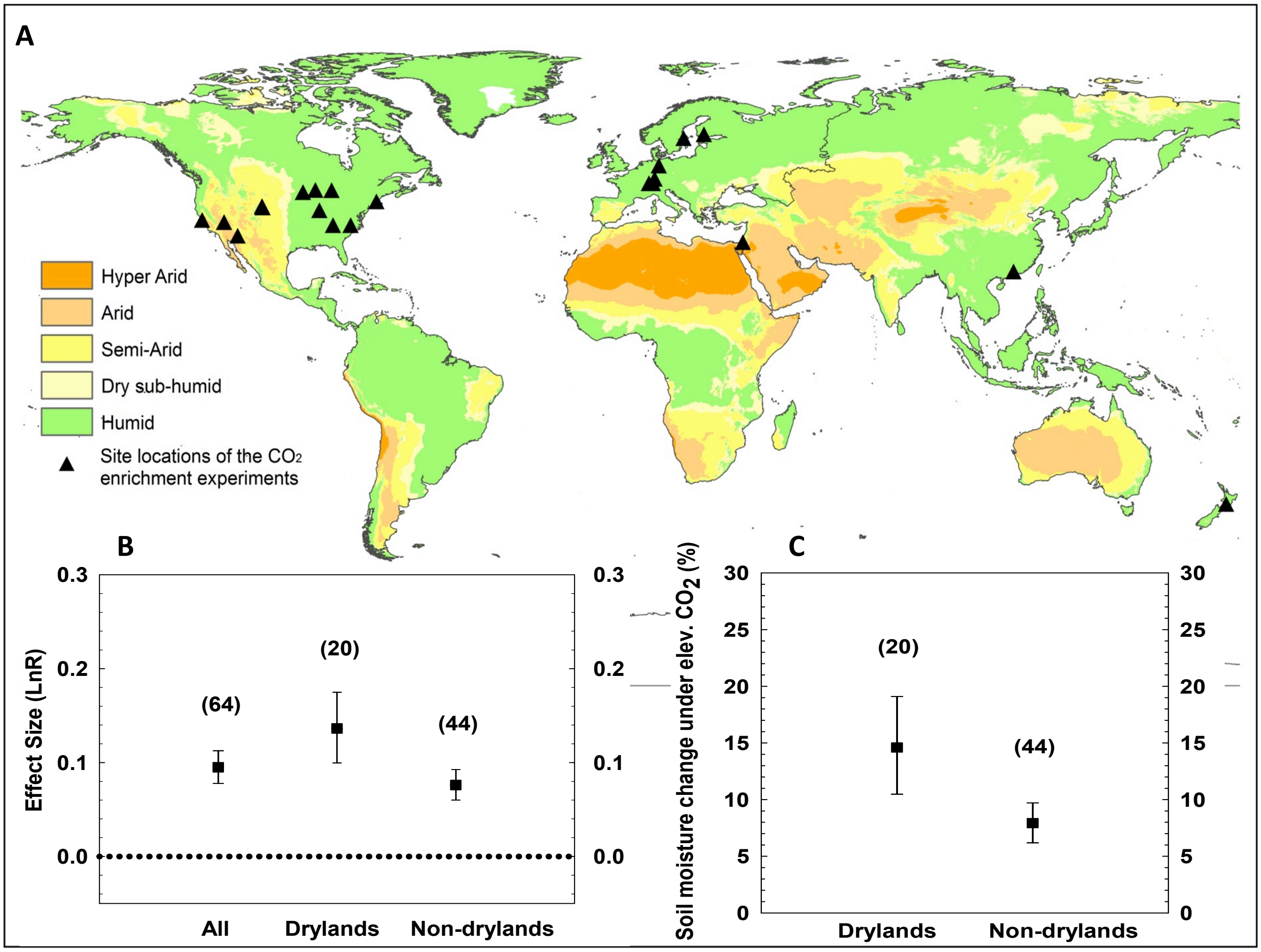

Figure S1. Comparisons of mean effect size and soil moisture response under elevated CO_2 for overall data (including both growing season data and non-growing season data). A. Site locations of the CO_2 enrichment experiments together with globally distributed climate zones based on a standard aridity index formulation (precipitation/potential evapotranspiration); B. Mean effect size of soil water content under elevated CO_2 for the entire data set, under dryland and non-dryland regimes. The effect size was calculated as the natural log of the magnitude of an experimental treatment mean (the soil water under elevated CO_2) relative to the control treatment mean (the soil water under ambient CO_2); C. Enhancement of soil water content under elevated CO_2 for dryland versus non-dryland regimes. The number of cases is shown in brackets. Error bars are bootstrapped confidence intervals (CI). All the statistics are significant at $P < 0.05$. The map was generated using ArcGIS for Desktop 10.3.1 (http://www.arcgis.com).

Table 1 A list of all the individual studies that were included in the meta-analysis. Only growing season data were used for the statistical analysis.
Soil Water Content Method: GSWC: Gravimetric Soil Water Content, VSWC: Volumetric Soil Water Content, PAW: Plant Available Water, WFPS: Water Filled Pore Space.
Vegetation Type: G: Grassland, F: Forest, C: Cropland. Soil Texture: S: Sand, LS: Loamy Sand, SL: Silt Loam, SCL: Silt/Sand Clay Loam, CL: Clay Loam, SDL: Sandy Loam, L: Loam, LC:Loamy Clay.

N°	Source	Location	SWC Method	Vegetation Type	Soil Texture	Soil Depth (cm)	CO_2 Enrich Factor	Sampling Time	Number of Data Points
1	Adair 2011	USA (45°00'N, 93°00'W)	VSWC	G	S	0-17	1.5	Jun - Oct 2006 (Continuous)	1
2	Allard 2005	New Zealand (40°14'S, 175°16'E)	VSWC	G	S	0-15	1.3	Dec 2000 - May 2001, Sept-Dec 2001 (Continuous)	1
3	Ambus 1999	USA (45°34'N, 84°40'W)	WFPS	F	LS	0-15	2	May 1, 28 & July 6	2[(1)]
4	Arnone 1998	Switzerland (47°28'N, 7°30'E)	GSWC	G	SCL	0-15	1.7	Springtime/Summer	2[(2)]
5	Bader 2009	Switzerland (47°28'N, 7°30'E)	VSWC	F	SCL	0-10	1.5	Apr - Oct 2007 (Continuous)	1
6	Baggs 2003	Switzerland (47°27'N, 8°41'E)	GSWC	G	CL	0-25	1.7	Jun-Oct 2000, Apr-Jun 2001 (Sampled twice per re-growth period)	1
7	Baggs 2004	Switzerland (47°27'N, 8°41'E)	GSWC	G	CL	0-25	1.7	Jun-Jul 2002 (Sampled periodically after fertilization)	1
8	Burkart 2004	Germany (52°18'N, 10°26'E)	PAW	C	LS	0-50	1.7	Apr – Aug 1998, 1999 (Continuous)	2[(3)]
9	Catovsky 1999	USA (42°30'N, 71°35'W)	GSWC	F	n/a	n/a	1.9	August 20-28 (Sampled every morning over 8-day watering cycle)	1
10	Carrillo 2011	USA (41°11'N, 104°54'W)	VSWC	G	L	0-20	1.7	Growing season in 2007, 2008 (Continuous)	1
11	Conley 2001	USA (33°06'N, 112°W)	VSWC	C	CL	0-180	1.5	Growing season in 1998, 1999 (Continuous)	2[(4)]
12	Craine 2001	USA (45°00'N, 93°00'W)	GSWC	G	S	0-20	1.5	Growing season in 1998, 1999	1
13	Decock 2012	USA (40°03'N, 88°12'W)	WFPS	C	SL	5-25	1.5	Growing season in 2005, 2006 (During each phenological stage)	1
14	Deng 2010	China (23°20'N, 113°30'E)	VSWC	F	SDL	0-5	1.9	Apr-Sept 2006, 2007, 2008 (Continuous)	1
15	Dijkstra 2010	USA (40°50'N, 104°42'W)	VSWC	G	L	0-20	1.5	Growing season in 2006 (Continuous)	1
16	Dijkstra 2013	USA (41°11′ N, 104°54′W)	WFPS	G	L	0-10	1.5	Growing season in 2007-2011 (Continuous)	1
17	Dubbs 2010	USA (35°58'N, 79°05'W)	VSWC	F	CL	0-30	1.5	Apr-Oct 2004, 2005, 2006 (Continuous)	1
18	Edwards 2001	New Zealand (40°14'S, 175°16'E)	VSWC	G	S	0-15	1.3	Nov 1998-May 1999, Sept-Dec 1999 (Continuous)	1
19	Ellsworth 1999	USA (35°58'N, 79°05'W)	VSWC	F	CL	0-30	1.5	June - Oct 2007 (Continuous)	1
20	Erbs 2009	Germany (48°42'N, 9°11'E)	VSWC	C	SL	0-30	1.4	May-Aug 2003, Mar-Aug 2004, 2005 (Continuous)	3[(5)]
21	Fatichi 2013	Switzerland (47°28' N, 7°30' E)	VSWC	F	LC	15-20	1.6	Summer 2004, 2005 (Continuous)	1
22	Field 1997	USA (37°24'N, 122°13'W)	VSWC	G	S	0-15	2	Apr-Jun 1995 (Continuous)	2[(6)]
23	Grunzweig 2001	Israel (31°21'N, 34°51'E)	GSWC	G	Bedrock	0-35	1.4/1.6	Mar-May 1997 (Measured weekly)	2[(7)]
24	Kettunen 2005	Finland (60°49′N, 23°30′E)	VSWC	C	Peat	n/a	2	2nd, 5th, 6th Period in May (Continuous)	1
25	Leakey 2006	USA (40°03′N, 88°12′W)	VSWC	C	SCL	5-25	1.5	May-Sept 2004 (Continuous)	1
26	Lecain 2003	USA (40°50'N, 104°42'W)	VSWC	G	SDL	0-100	2	Growing season in 1998, 2000 (Continuous)	1
27	Lecain 2012	USA (41°11'N, 104°54'W)	VSWC	G	SDL	n/a	1.9	Growing season (Continuous)	3[(8)]
28	Leuzinger 2007	Switzerland (47°28'N, 7°30'E)	VSWC	F	LC	0-90	1.5	Jun-Jul 2004, Jul-Aug 2005	2[(9)]
29	Liu 2008	China (23°20'N, 113°30'E)	VSWC	F	SDL	0-70	1.9	Jun-Sept 2006, Jan-Mar 2007 (Continuous)	2[(10)]
30	Marhan 2010	Germany (48°42'N, 9°11'E)	VSWC	C	SL	0-15	1.4	Mar-Oct 2003-2006 (Continuous)	1
31	Matamala 2000	USA (35°58'N, 79°05'W)	VSWC	F	CL	0-30	1.5	May-Oct 1997, Apr-Oct 1998 (Continuous)	1
32	McCarthy 2010	USA (35°58'N, 79°05'W)	VSWC	F	CL	0-30	1.5	May-Oct 1997, Apr-Oct 1998-2004 (Continuous)	1
33	McLain 2008	USA (35°58'N, 79°05'W)	VSWC	F	CL	0-30	1.5	Apr-Oct 2000 (Continuous)	1
34	Morgan 2001	USA (40°40'N, 104°45'W)	VSWC	G	SDL	n/a	2	Growing season in 1997, 1998 (Continuous)	1
35	Mosier 2002	USA (40°50'N, 104°42'W)	VSWC	G	SDL	0-15	1.9	Apr-Oct 1997-2000 (Continuous)	2[(11)]
36	Nelson 2004	USA (40°49' N, 107°47' W)	VSWC	G	SDL	0-105	2	Apr-Oct 1997-2001 (Continuous)	1
37	Nendel 2009	Germany (52°18'N 10°26'E)	VSWC	C	LS	0-30	1.5	Growing season (Continuous)	1
38	Newton 2003	New Zealand (40°14'S, 175°16'E)	VSWC	G	S	0-15	1.3	Sept-May 1998-2003 (Continuous)	1
39	Niklaus 1998	Switzerland (47°33'N 7°34'E)	VSWC	G	L	0-10	1.7	Mar, Jun, Oct 1995, 1996 (Continuous)	1
40	Pataki 2000	USA (36°49′N, 115°55′W)	VSWC	G	LS	0-20	1.9	Oct-Nov 1998 (Continuous)	1
41	Pendall 2003	USA (40°40′N, 104°45′W)	VSWC	G	SDL	0-15	2	Apr-Oct 1999, 2000 (Continuous)	1
42	Pregitzer 2006	USA (49°40′N, 89°37′E)	VSWC	F	SDL	0-10	1.5	Growing seasons in 2002-2004 (Continuous)	1
43	Sindhoj 2000	Sweden (59°48′N, 17°38′E)	VSWC	G	CL	0-25	1.9	Growing season in 1997, 1998 (Continuous)	1
44	Volk 2000	Switzerland (47°33′N, 7°34′E)	GSWC	G	SCL	0-25	1.7	June - July 2007	1
45	Wall 2001	USA (33.1°N, 112.0°W)	VSWC	C	CL	0-180	1.5	Growing season (Continuous)	1

**Total Studies**	**45**
**Total Data Points**	**58**

[(1)] The soil was incubated with low nitrogen and 'high' nitrogen treatment
[(3)] [(4)] Crops were treated with two irrigation regimes - well watered vs. drought stress
[(7)] The experiment was conducted at two different soil texture – rocks vs. sand
[(6)] FACE experiment was taken at two CO_2 enrichment factors: 1.4 and 1.6
[(2)] [(5)] [(8)] [(10)] [(11)] Plants are subject to different climatic regimes
[(9)] Grassland is treated with nitrogen addition and defoliation or none
